# Age-related differences in resting state functional connectivity in pediatric migraine

**DOI:** 10.1186/s10194-021-01274-y

**Published:** 2021-07-06

**Authors:** Tiffany Bell, Akashroop Khaira, Mehak Stokoe, Megan Webb, Melanie Noel, Farnaz Amoozegar, Ashley D. Harris

**Affiliations:** 1 Department of Radiology, University of Calgary, Calgary, AB Canada; 2 Hotchkiss Brain Institute, University of Calgary, Calgary, AB Canada; 3 Alberta Children’s Hospital Research Institute, University of Calgary, Calgary, AB Canada; 4 Department of Psychology, University of Calgary, Calgary, AB Canada; 5 Department of Clinical Neurosciences, University of Calgary, Calgary, AB Canada

**Keywords:** Migraine, pediatric, resting-state functional connectivity, power spectra, brain development, Independent Component Analysis

## Abstract

**Background:**

Migraine affects roughly 10% of youth aged 5–15 years, however the underlying mechanisms of migraine in youth are poorly understood. Multiple structural and functional alterations have been shown in the brains of adult migraine sufferers. This study aims to investigate the effects of migraine on resting-state functional connectivity during the period of transition from childhood to adolescence, a critical period of brain development and the time when rates of pediatric chronic pain spikes.

**Methods:**

Using independent component analysis, we compared resting state network spatial maps and power spectra between youth with migraine aged 7–15 and age-matched controls. Statistical comparisons were conducted using a MANCOVA analysis.

**Results:**

We show (1) group by age interaction effects on connectivity in the visual and salience networks, group by sex interaction effects on connectivity in the default mode network and group by pubertal status interaction effects on connectivity in visual and frontal parietal networks, and (2) relationships between connectivity in the visual networks and the migraine cycle, and age by cycle interaction effects on connectivity in the visual, default mode and sensorimotor networks.

**Conclusions:**

We demonstrate that brain alterations begin early in youth with migraine and are modulated by development. This highlights the need for further study into the neural mechanisms of migraine in youth specifically, to aid in the development of more effective treatments.

## Introduction

Migraine is a chronic condition characterised by recurrent, severe headaches. Migraine is one of the top five most frequent childhood diseases, affecting over 10% of youth aged 5–15 [1]. Neuroimaging studies have shown structural and functional differences in the brains of migraine sufferers [2–4]. Whether these differences are a cause or a consequence of repeated migraine attacks is unclear, although decreases in grey matter have been seen in youth with migraine aged 9–17 [5].

Children and adolescents with migraine show differences in resting-state functional connectivity in the brain. Resting-state functional connectivity analysis focuses on spontaneous Blood Oxygen Level Dependent (BOLD) signal oscillations which occur during the absence of a stimulus/task. Areas of the brain where the BOLD signal oscillations are correlated are interpreted to be functionally connected, generating spatial maps of functional connectivity. Using independent component analysis (ICA), Colon et al. (2019) showed significant differences in intra-network connectivity in sensory and cognitive networks in adolescents with migraine aged 12–19 [6]. Also using ICA, Messina et al. (2019) found both altered intra- and inter-network connectivity in brain networks involved in multisensory processing and cognitive control of pain in youth with migraine aged 9–17 [7].

Although both studies provide important insight into connectivity differences in youth with migraine, these studies encompass an age range in which the brain is changing dramatically; during adolescence grey matter volume decreases, whilst white matter increases, representing synaptic pruning and a shift from local to distal connectivity profiles. These changes are modulated by puberty, with reductions in grey matter occurring with increasing pubertal stage and earlier pubertal timing [8], and increases in white matter associated with pubertal stage [9]. Not only is adolescence a pivotal period for brain development, but it is also a transitionary time for migraine. Migraine prevalence increases with increasing age in children and adolescents [10]. During puberty, the prevalence of migraine changes from a 1:1 male to female ratio in childhood to a 1:2 male to female ratio in adulthood [11]. Therefore, it is highly likely there are sex and puberty specific changes in the migraine brain. Indeed, Faria et al. (2015) used seed-based analysis to investigate sex differences in the brain of youth with migraine. They found increased connectivity between the amygdala and precuneus (seed regions) and brain regions associated with sensory, motor and affective function in females with migraine aged 10–16 compared to males and healthy controls. Additionally, they split their cohorts into two groups based on age (10–11 years and 15–16 years) to investigate the impact of development on sex differences. Though they found structural differences using this analysis, no connectivity differences were seen [12]. However, this null effect of age is limited to areas connected to the amygdala and precuneus seed regions. Additionally, no specific measure of puberty was included.

In addition to investigating spatial maps of functional connectivity, multiple studies have demonstrated that investigating the frequency of BOLD oscillations (quantified using power spectra analyses) within these spatial maps reveals novel information regarding brain organisation and dynamics [13–15]. As well as analysing data from the frequencies typically filtered out in resting-state analysis, analysis of BOLD oscillation frequency extends the typical correlation-based analysis of resting-state data to provide information on BOLD dynamics themselves. The exact functional relevance of changes in these dynamics is unknown, but has been suggested to reflect alterations in connectivity and information processing [16, 17]. The dominant activity of brain regions follows a functional hierarchy, with rapid dynamics in sensory regions and slower oscillations in regions associated with integrative processes [17]. Additionally, functional connectivity within and between networks has been shown to be controlled in frequency specific manners [18] providing insight to the interpretation and relevance of these measures. Changes in BOLD oscillation frequency have been shown in multiple disorders, including major depressive disorder [16], idiopathic generalised epilepsy [19], schizophrenia [20] and chronic back pain [21]. To our knowledge, frequency analysis has not been performed to investigate migraine in children, despite the striking alterations in frequency profiles seen in chronic pain [21].

The aim of the present study was to investigate resting-state functional connectivity in migraine during the period of transition from childhood to adolescence. We used both spatial ICA and power spectra analyses to compare resting-state networks between youth aged 7–15 years with and without migraine, with a focus on the effects of age, sex and pubertal status. This study builds on previous work by including a younger age range, a specific measure of pubertal status and spectral frequency analysis. Additionally, as there is evidence that changes in the brains of migraine sufferers are related to the time since their last migraine [22–25], we explored the association between resting-state connectivity and the position of the child in the migraine cycle to provide new insight into the dynamics of functional connectivity across the migraine cycle.

## Materials and methods

### Participants

Twenty-seven youth aged 7–15 years who had previously received a diagnosis of migraine from their family physician were recruited from a tertiary-level chronic pain program and the surrounding community. Participants were included if they had a physician diagnosis of migraine, which was confirmed using the International Classification of Headache Disorders 3rd edition (ICHD-III) beta diagnostic criteria [26], had no other accompanying neurological, psychiatric or neurodevelopmental disorders (e.g. attention deficit hyperactivity disorder (ADHD), Autism), met the standard magnetic resonance imaging (MRI) safety criteria (e.g. no metal implants or devices) and were not taking medications that could interfere with brain chemistry, such as triptans.

Twenty-two age- and sex-matched children without migraine (control group) were recruited using the Healthy Infants and Children Clinical Research Program (HICCUP). The same exclusion criteria of no neurological, psychiatric or neurodevelopmental disorders and standard MRI safety criteria were applied for the control participants; additionally, control participants were excluded if they had any history of migraine or other headache disorder.

### Migraine diary

The parents of the youth with migraine were asked to keep an electronic migraine diary for 30 days preceding their appointment and 7 days following. In this diary, they were asked to record if their child had had a migraine that day. If so, they were asked to record the length of the migraine attack and the pain level (using the Wong-Baker FACES pain rating scale of 1–10), along with how they treated the migraine. If the child did not have a migraine in the 7 days following the appointment, they were asked to provide the date of the next following migraine. Youth with migraine were excluded if they did not experience a migraine in the 30 days preceding their appointment, or if they experienced a migraine on the day of the appointment. A metric for the position in the migraine cycle was calculated by using the following equation:

$$ \mathrm{migraine}\ \mathrm{cycle}\ \mathrm{metric}=\frac{\mathrm{days}\ \mathrm{since}\ \mathrm{last}\ \mathrm{migraine}}{\left(30/\mathrm{number}\ \mathrm{of}\ \mathrm{migraine}\mathrm{s}\ \mathrm{in}\ 30\ \mathrm{days}\right)} $$

This metric represents the time since their last migraine normalised by frequency. A higher value indicates they are further along in their migraine cycle (or closer to the onset of their next migraine).

### Questionnaires

All participants completed the following psychometrically-sound questionnaires: Headache Impact Test (HIT-6) [27], Pediatric Migraine Disability Assessment (PedMIDAS) [28], Revised Children’s Anxiety and Depression Scale (RCADS) short version [29], and the Puberty Status Scale [30]. The HIT-6 is a six-item questionnaire which assesses the negative impact of headaches on normal daily activities in the general headache population. Scores range from 36 to 78, with higher scores representing higher negative impact [27, 31]. The PedMIDAS is a six-item questionnaire which assesses headache-related disability, designed for, validated and shown to be reliable in children and adolescents. Scores range from 0 to 90, with higher scores representing higher headache impact [28]. The RCADS short form is a 24-item questionnaire which assesses symptoms of anxiety and depression based on diagnostic criteria from the Diagnostic and Statistical Manual of Mental Disorders, fourth edition (DSM-IV). This questionnaire was designed for, validated and shown to be reliable in children and adolescents [29, 32]. Scores range from 0 to 75, higher scores indicate stronger symptoms. The pubertal development scale is a 5-item questionnaire based on the Tanner pubertal development scale. Responses can be grouped into the following categories: prepubertal, early puberty, mid-pubertal, late puberty and post-pubertal. This questionnaire has been shown to be valid and reliable [30]. Due to the relatively small number of children recruited scoring mid and late puberty, these categories were grouped together and classed as “mid-late puberty”.

### MRI acquisition

Scanning was performed on a 3 T GE 750w MR scanner using a 32-channel head coil. A T1-weighted anatomical image was collected for registration (BRAVO; TE/TR = 2.7/7.4 ms, slice = 1 mm^3^ isotropic voxels). Resting state data was collected using a T2*-weighted echo planar imaging sequence (165 volumes, TE/TR = 29/2500 ms, slice thickness = 3.5 mm, matrix size = 64 × 64, FOV = 22 cm^3^). During the scan, subjects watched INSCAPES [33], and were instructed to keep their eyes open and try not to think of anything in particular. INSCAPES is a movie designed to provide sufficient stimulation to reduce movement and increase wakefulness in the scanner whilst minimising cognitive load. INSCAPES has been shown to evoke an intermediate level of stimulus-evoked information processing in healthy controls, less so than a movie but more so than rest. For example, measures of connectivity measured at rest correlated more strongly with connectivity measured watching INSCAPES than with connectivity measured watching a movie [33].

### MRI analysis

Data were preprocessed using FSL [34] which consisted of the following steps: brain extraction (BET) [35], motion correction (MCFLIRT) [36], slice timing correction, spatial smoothing (6.0 mm) and high pass filtering (100 s). Subjects were excluded if peak motion was higher than 5 mm. Single subject ICA was run on the preprocessed data and noise components were removed using the criteria specified in Ref [37].

ICA was performed using the Group ICA of fMRI toolbox (GIFT) (GroupICATv4.0b; https://trendscenter.org/software/gift/), which consists of four main steps: 1) concatenation of all subject scans 2) data reduction 3) group ICA, and 4) back reconstruction to estimate subject-specific spatial maps and time courses. Group ICA using all participants was performed using the infomax algorithm with the number of components limited to 30. Stability was assessed using ICASSO [38] with 20 repetitions. Components were rejected if the stability metric was under 0.8 (1 component). After back reconstruction, the spatial components were converted to z-scores. The 29 remaining components were visually inspected and labelled based on previously reported resting-state networks [39–41]. Components in which the signal was not primarily localised in grey matter and the timeseries was not dominated by low frequency oscillations were labelled as noise and discarded. For each network component, two subject-specific outcome measures were obtained: (1) spatial maps thresholded based on the distribution of voxel wise t-statistics and (2) power spectra estimated on the detrended component time courses. These two measures represent intra-network connectivity. In addition, subject-specific connectivity between spatial maps (internetwork connectivity) was estimated using Pearson’s correlation coefficient, transformed to z-scores using Fisher’s transformation [42].

### Statistical analysis

Statistical comparisons were performed using the MANCOVAN toolbox implemented in GIFT [42]. Separate analysis of covariance were conducted on the measures of intra (spatial maps and power spectra) and internetwork connectivity with the following predictors: (1a) group (migraine or control) and age, (1b) group, and sex and (2) migraine cycle metric (migraine group only) and age. Group by pubertal status comparisons were included as a secondary exploratory analyses. Significance for univariate tests was set at *p* < 0.05 and all results are corrected for multiple comparisons using FDR correction. None of the comparisons showed statistical differences in internetwork connectivity, and therefore this is not reported further.

## Results

Participant demographic data and questionnaire results are shown in Table 1. Of the 49 children from which imaging data was acquired, 9 children were excluded due to excessive motion (> 5 mm displacement, 4 from the migraine group and 5 from the control group), 4 children from the migraine group were excluded due to signal drop out, one child was excluded as they experienced a migraine on the day of the session, two children were excluded as they did not have a migraine in the 30 days prior to the scan. This left 16 children in the migraine group and 17 children in the control group. In the migraine group, 13 were classified as having episodic migraine (< 15 per month) and 3 were classified as having chronic migraine (> 15 per month). Eight suffered from visual aura, 7 had no aura symptoms and 1 did not specify.

**Table 1:** Table of demographics and questionnaire results (mean ± standard deviation)

	Migraine	Control	Comparison
N	16 (7 female)	17 (11 female)	X^2^(1,N = 33) = 1.460, *p* = 0.227
Age in years	11.3 (2.0)	10.9 (1.4)	t(30) = − 0.795, *p* = 0.486
(Age range in years)	8.3–15.3	8.6–14.0	
HIT-6	**60.4 (6.7)**	**43.2 (3.2)**	**F(1,31) = 90.150,** ***p*** **< 0.001**
PedMIDAS	**21.4 (21.6)**	**1.1 (1.4)**	**F(1,31) = 14.282,** ***p*** **= 0.001**
RCADS Total	19.1 (10.0)	13.8 (7.1)	F(1,31) = 1.671, *p* = 0.206
Pubertal Status (N)			
**Pre**	8	4	X^2^(3,*N* = 33) = 2.639, *p* = 0.267
**Early**	5	7	
**Mid-Late**	3	6	
Number of Migraines in 30 days	7.4 (8.5)	N/A	
Range of number of migraines in 30 days	1–30	N/A	
Disease Duration (years)	4.4 (3.2)	N/A	N/A
Mean Displacement (mm)	1.34 (1.23)	0.77 (0.56)	t(21)^a^= − 1.702, *p* = 0.104

*HIT-6* Headache Impact Test, *PedMIDAS* Pediatric Migraine Disability Assessment, *RCADS* Revised Children’s Anxiety and Depression Scale

^a^Equal variances not assumed

**Fig. 1:**
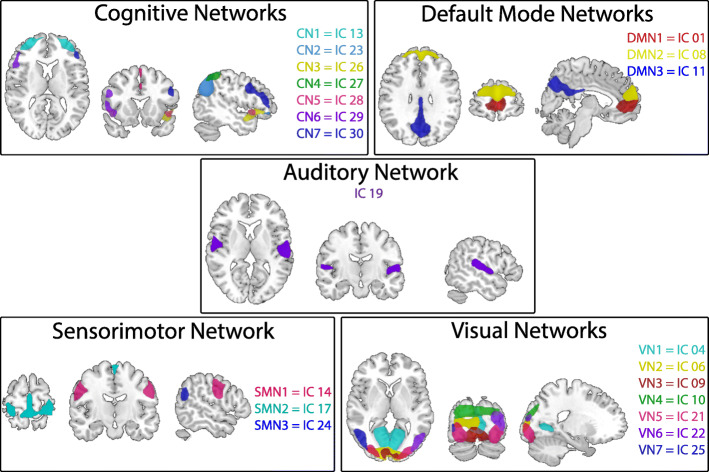
Spatial maps of the 19 independent components (IC) identified as resting-state networks, grouped with their respective networks based on anatomical location. Components are plotted as z-statistics, thresholded at 3.0. CN: Cognitive Network; DMN: Default Mode Network; SMN: Sensorimotor Network; VN: Visual Network

Of the 30 components, 19 components were identified as part of resting state networks (Fig. 1) and 11 were identified as noise.

### Effects of age, sex and pubertal status on group differences in connectivity

**Fig. 2:**
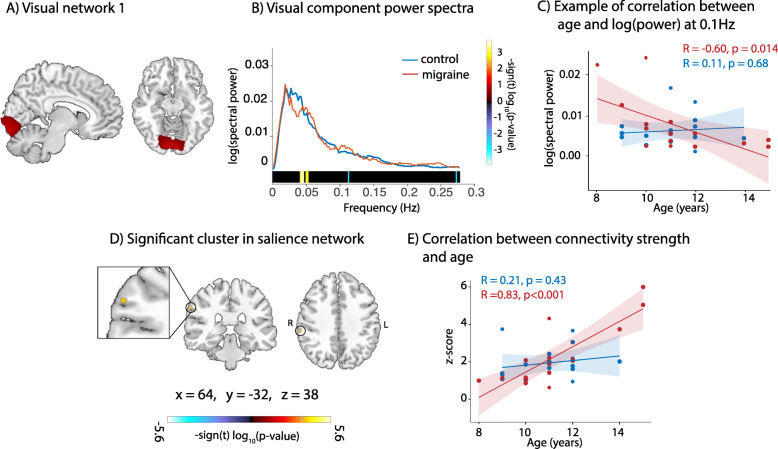
Group by age interaction effects on connectivity. **a** Visual network 1. **b** Power spectra from visual network 1. Frequencies where significant group by age interactions were present are highlighted at the bottom. **c** Correlation between log (spectral power) and age per group at 0.1 Hz **d** Cluster in the supramarginal gyrus exhibiting significant group by age interaction effects on connectivity within the salience network (number of voxels = 11) **e** Correlation between connectivity strength in the supramarginal gyrus and age per group. The migraine group is represented in red and controls in blue for b, c, and e

There was no significant effect of group alone on functional connectivity, however there were significant group by age interactions. Power spectra analysis showed that, in visual network 1 (Fig. 2A) of children with migraine, there was a significant positive relationship between the power of low frequency oscillations and age (highlighted in yellow in Fig. 2B), and a significant negative relationship between the power of higher frequency oscillations and age (highlighted in blue in Fig. 2B and visualised in Fig. 2C): older children with migraine had more low frequency oscillations and less high frequency oscillations than younger children with migraine. Neither of these relationships were present in controls.

Spatial map analysis shows a significant group by age interaction present in connectivity of the supramarginal gyrus within the salience network (Fig. 2D). There was a significant positive correlation between connectivity and age in children with migraine that was not present in control children (visualised in Fig. 2E). Older children with migraine had stronger connectivity of the supramarginal gyrus to the salience network than younger children with migraine.

**Fig. 3:**
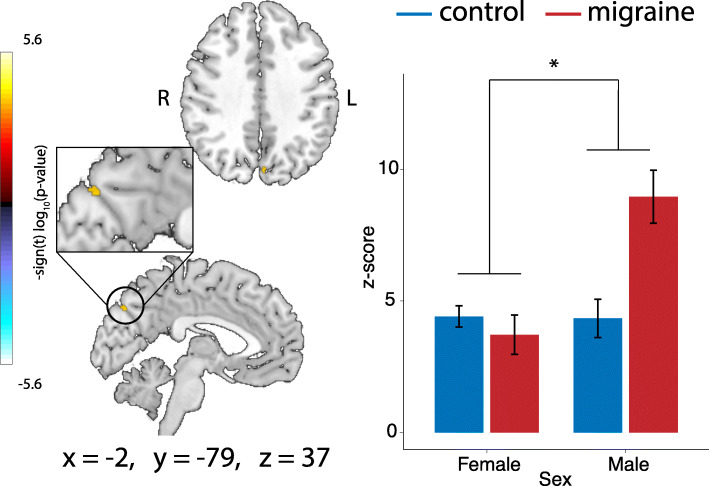
Group by sex interaction effects on connectivity. Cluster in the precuneus exhibiting significant group by gender interaction effects of connectivity with the default mode network (number of voxels = 11) and connectivity values per group. *p < 0.05

There was a significant group by sex interaction present in connectivity of the precuneus within the default mode network (Fig. 3). Males with migraine had stronger connectivity in the posterior cingulate cortex within the default mode network compared to control males, whereas there was no difference in connectivity between females with and without migraine.

**Fig. 4:**
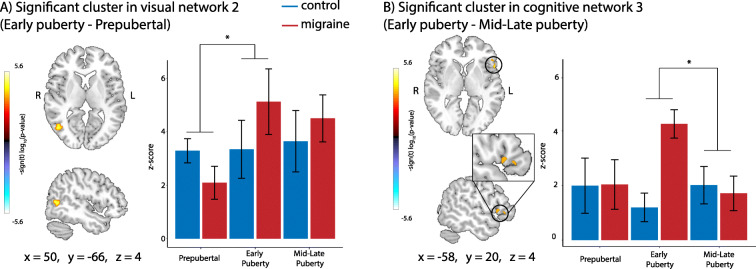
Group by pubertal status interaction effects on connectivity. **a** Cluster in the lateral occipital cortex exhibiting significant group by pubertal status interaction effects on connectivity within visual network 2 (number of voxels = 137), and connectivity values per group. **b** Cluster in the inferior frontal gyrus exhibiting significant group by pubertal status interaction effects on connectivity within cognitive network 3 (number of voxels = 45), and connectivity values per group. *p < 0.05

In a secondary, exploratory analysis, we looked at the interactions between group and pubertal status. There was a significant group by pubertal status interaction effect on connectivity. Prepubertal children with migraine had weaker connectivity in the lateral occipital cortex within visual network 2 than control children, however children with migraine in early puberty had stronger connectivity compared to control children (Fig. 4). Children with migraine in early puberty had stronger connectivity in the inferior frontal gyrus within cognitive network 3 (frontal parietal network) compared to control children, whereas there was no difference between control children and children with migraine in mid-late puberty (Fig. 4B).

### Effects of the migraine cycle on connectivity

**Fig. 5:**
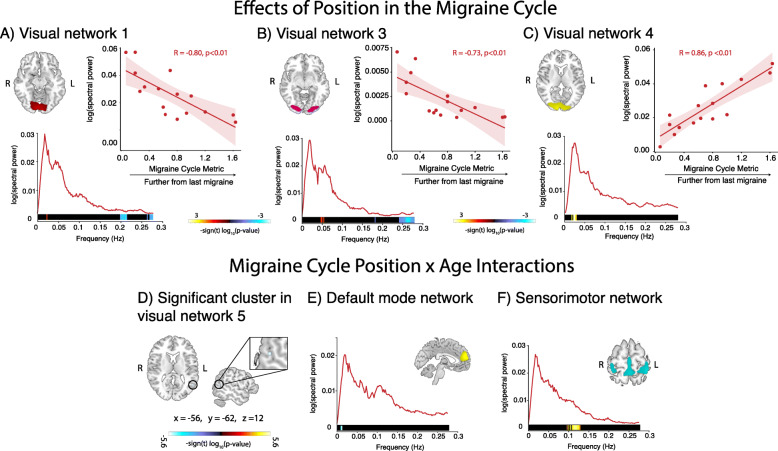
Location in the migraine cycle and location by age interaction effects on connectivity. **a** Power spectra from visual network 1, frequencies which significantly correlated with the migraine cycle metric are highlighted at the bottom. Example of negative correlation between log (spectral power) at 0.20 Hz and position in the migraine cycle is shown. **b** Power spectra from visual network 3, frequencies which significantly correlated with migraine cycle metric are highlighted at the bottom. Example of negative correlation between log (spectral power) at 0.26 Hz and position in the migraine cycle is shown. **c** Power spectra from visual network 4, frequencies which significantly correlated with migraine cycle metric are highlighted at the bottom. Example of positive correlation between log (spectral power) at 0.02 Hz and position in the migraine cycle is shown. **d** Cluster in the lateral occipital cortex exhibiting significant group by pubertal status interaction effects on connectivity within visual network 5 (number of voxels = 11). **e** Power spectra from the default mode network, frequencies where significant position by age interactions were present are highlighted at the bottom. **f** Power spectra from the sensorimotor network, freqeuncies where significant position by age interactions were present are highlighted at the bottom

There was a significant positive correlation between the power of low frequency oscillations in visual components 1, 3 and 4 and the migraine cycle metric (highlighted in red/yellow at the bottom of the power spectra in Fig. 5A, B and C) and a significant negative correlation between the power of high frequency oscillations in visual components 1 and 3 and the migraine cycle metric (highlighted in blue at the bottom of the power spectra in Fig. 5A and B). Children who were further along in their migraine cycle had more low frequency activity and less high frequency activity in the visual networks. There was also a significant age by migraine cycle metric interaction in the lateral occipital cortex within visual component 5 (Fig. 5D), in low frequency oscillations (0.01 Hz) within the default mode network (Fig. 5E) and high frequency oscillations (~ 0.1 Hz) in the sensorimotor network (Fig. 5F).

## Discussion

In this study, we compared resting-state network connectivity in youth with migraine aged 7–15 years and age-matched controls. Our main findings can be summarised as: (1) significant group by age interactions in the visual and salience networks, group by sex interactions in the default mode network and group by pubertal status interactions in visual and frontal parietal networks; (2) significant relationships between connectivity in the visual networks and position in the migraine cycle, and age by cycle position interactions in the visual, default mode and sensorimotor networks and (3) no group differences in internetwork connectivity.

### Group differences in connectivity are modulated by age, sex and pubertal status

Compared to older children with migraine, younger children with migraine had less low and more high frequency oscillations in visual network 1, likely reflecting altered connectivity [20]. Younger children also showed weaker connectivity of the supramarginal gyrus within the salience network (cognitive network 5). This relationship between connectivity and age was not present in controls. These connectivity differences in the visual and salience networks may reflect neurophysiological changes in migraine. Indeed, transcranial magnetic stimulation (TMS) measures have shown hyperexcitability in the visual cortex of migraine suffers [25]. The salience network comprises core regions involved in pain processing such as the primary and secondary somatosensory, cingulate and insular cortices [43]. The supramarginal gyrus in particular is involved in the interpretation of somatosensory data [44, 45]. Common symptoms in migraine are allodynia [46] and increased pain sensitivity [47]. The connectivity changes in the supramarginal gyrus found here may reflect alterations in sensory processing and could be related to altered pain sensitivity.

Males with migraine had stronger connectivity in the precuneus within the default mode network compared to control males, whereas there was no difference in connectivity between females with and without migraine. Within the default mode network, adults with migraine have been shown to have higher connectivity of the precuneus compared to controls [6, 48], and connectivity of this region positively correlated with the HIT-6 score [48]. In contrast, one study showed adolescents with migraine had decreased connectivity in the left parieto-occipital junction of the default mode network compared to healthy controls [7], and another study found no differences in connectivity in the posterior portion of the default mode network between adolescents with migraine and controls [6]. These discrepancies may be due to the sex effects on connectivity shown here. Though sex differences have been reported in default mode network connectivity in healthy children previously [42, 49], there have been no previous studies investigating sex differences in default mode network connectivity in youth with migraine. This is a particularly interesting question given the gender specific trajectories of default mode connectivity and migraine development. In a sample of healthy adolescents aged 13–15 years, females showed stronger within network connectivity than males, and connectivity increased with pubertal maturation in females but decreased with pubertal maturation in males [49]. As our current results show males with migraine have higher default mode network connectivity compared to both control males and females, we suggest that, for males who transition out of migraine, this increased connectivity decreases. For males who continue to have migraines, this increased connectivity remains high. By contrast, we suggest that connectivity increases rapidly and is more pronounced in females with migraine across adolescence compared to controls. This hypothesis can be best examined using a longitudinal study.

We found significant group by pubertal status interaction effects on connectivity within the visual and control networks, though it should be noted that the number of subjects in each pubertal group was low, and therefore these results warrant further exploration. Within visual network 2, prepubertal children with migraine had weaker connectivity of the lateral occipital cortex compared to control children, however during early puberty, children with migraine had stronger connectivity compared to control children. This suggests that visual network connectivity differences in migraine begin early in development, followed by a dramatic increase in connectivity in children with migraine as they enter puberty. Within the frontal parietal network (cognitive network 3), children with migraine in early puberty had stronger connectivity of the inferior frontal gyrus, however children with migraine in mid-late puberty had weaker connectivity in this area as compared to control children. This implies a dramatic increase of connectivity in early puberty (similar to the previous finding), which then decreases during mid-late puberty. The inferior frontal gyrus is involved in pain processing and is thought to play a role in the anticipation of, and attention to, pain [50], and is one of several areas of reduced grey matter volume in adult [3] and pediatric migraine sufferers aged 9–17 [5]. Functional connectivity in these networks has been previously shown to be altered in children [6, 7] and adults [51–54] with migraine. The results presented here suggest there may be interaction effects of sex and pubertal status on broader connectivity, and this should be further explored in future studies using a longitudinal design.

Indeed, there is evidence that children with migraine have abnormal brain maturation. Contingent negative variation, a measure of cortical excitability, has been shown to decrease from childhood to adulthood in healthy controls and children with migraine who went into remission, however this decrease was substantially lower in children who continued to suffer from migraine [55]. Additionally, children with migraine have been shown to demonstrate higher amplitude of the N140 somatosensory evoked potential during selective attention as compared to a neutral condition, but this difference was not seen in control children. As this increase in amplitude during selective attention is typically seen in adult data, the authors speculate that the presence of this change in children with migraine but not control children represents earlier maturation of frontal connections [56]. In contrast, whilst healthy controls showed N135 latency reduction with age in high spatial frequency, children with migraine showed N135 latency reduction with age in lower spatial frequencies, which the authors suggest represents a lack of visual system maturation in children with migraine [57]. These findings indicate non-global effects on migraine brain development, supporting our regionally specific findings and our hypothesis of developmental alterations during migraine. This further highlights the need for early identification and treatment of migraine in childhood.

The changing pattern of headache typically seen in childhood migraine further supports the notion of migraine being a progressive, developmental disorder. In a longitudinal study, Virtanen et al. found that roughly 50% of children who suffered from migraine at age 6 had experienced changes in their headache profile by age 13 [58]. Indeed, chronic migraine is rarely the first presentation, instead it evolves from episodic migraine with gradual increases in attack frequency, indeed ineffective treatment of episodic migraine is one of the major risk factors for migraine chronification [59]. In this study, we chose to include both episodic and chronic migraine sufferers to represent the full spectrum of migraine patients. Episodic and chronic migraine are often considered two ends of the same spectrum and therefore the inclusion of both should highlight headache frequency specific changes. However, the majority of subjects were episodic sufferers, limiting the conclusions that can be drawn. Future studies would benefit from comparing episodic and chronic sufferers, to determine changes with migraine chronification.

### Connectivity in the visual cortex is related to the time since the last migraine attack

Within the migraine group, we also found a relationship between connectivity and the position in the migraine cycle; a metric that enables comparison of brain differences related to time from last migraine attack, while controlling for differences in migraine frequency. Children who are further along in their migraine cycle (i.e., more time from their previous attack) have more low and less high frequency oscillations in the visual networks. We also found a significant interaction between age, position in the migraine cycle and connectivity.

Studies which have scanned migraine patients repeatedly throughout the month have shown altered hypothalamic activation in response to stimuli 2 days before an attack [60, 61] and altered resting connectivity of the nucleus accumbens 3 days before a migraine attack [60]. Our findings likely differ due to the methods used, Schulte et al. used ROI-ROI analysis to measure connectivity, rather than the ICA approach used here. Indeed, previous TMS studies have shown changes in cortical excitability in the sensorimotor [23, 24] and visual [25] cortices as the migraine cycle progresses, which may be reflected as changes in spectral power and functional connectivity. We have also recently shown a negative correlation between levels of GABA in the thalamus and the position of the child in their migraine cycle [22]. As the thalamus is the gate for sensory and visual information [62], changes in the thalamus may lead to changes in functional connectivity in sensory and visual networks.

### No differences were seen in internetwork connectivity

In contrast to Messina et al. (2019), we found no differences in internetwork connectivity. We speculate that this is a function of the different ages included in each study. Between ages 9–11 years, substantial maturational changes occur in the brain. For example, grey matter levels likely peak at around 9–11 years of age, whereas white matter levels continue to increase throughout adolescence [63]. This is thought to represent a shift from segmented cognitive modules to interactions between hubs [64], allowing recruitment of higher order association areas to support higher cognitive skills and suppression of irrelevant sensory processes [63]. Half of the current study cohort (< 11 years) were just reaching peak grey matter levels. By contrast, the cohort sampled in Messina et al. (mean age 13 years) were in the development phase with predominant changes in distal connectivity rather than local grey matter development.

### Spectral ICA reveals novel information about BOLD oscillations in migraine

A unique component of this study is the use of power spectra as a measure of connectivity. Studies have shown that the frequency of BOLD oscillations reveals novel information regarding functional organisation of the brain, and changes in BOLD oscillation frequency have been shown in multiple disorders [16, 19–21]. Here, we show an age dependent change in BOLD signal oscillations in the visual network of children with migraine and an association between BOLD signal oscillations and the migraine cycle metric in multiple visual networks. The functional relevance of these changes in BOLD frequency oscillations is not fully understood but may reflect a disruption of resting-state dynamics associated with migraine.

Garrity et al. hypothesised that a shift from lower to higher frequency oscillations reflects a lower degree of connectivity [20]. However, Baliki et al. showed adult chronic back pain patients have both greater high frequency power in the medial prefrontal cortex compared to controls and increased connectivity of the medial prefrontal cortex with other pain-related regions [21]. The shift in power with age seen in the visual network of children with migraine may therefore reflect a mechanism for changes in brain connectivity specific to migraine development. There is a spatial distribution of power for BOLD oscillations; low frequencies show the highest power in the frontal, parietal and occipital cortices while high frequencies show the highest power in regions associated with more complex information processing such as the cingulate, temporal cortices and subcortical areas [15]. The observation of higher frequency power in the visual cortex of younger children in the migraine group may represent an increase in connectivity with more salient areas, perhaps related to an increased attentional state. Indeed, there is evidence of an attention bias in youth with chronic pain [65], and adults with migraine are thought to be hyperresponsive to stimuli due to increased attentional processes [66].

As children moved further from their last migraine (and approached the next), spectral frequency in the visual networks decreased. This may represent an increase in neural synchrony, as adults with migraine have been shown to have an increase in mean phase synchronisation measured using electroencephalography (EEG) in the presence of visual stimuli, whereas this synchronisation decreased in controls [67]. Additionally, adults with migraine have been shown to have altered photic drive (the tendency of cortical neurons to synchronise their frequency to visual stimuli) compared to controls, and the photic drive fluctuates throughout the migraine cycle [68–70]. However, as EEG measures frequency on a much faster scale than BOLD spectral analysis, this interpretation should be considered with caution.

### Naturalistic viewing in migraine

During the scan, participants in this study watched INSCAPES [33], a movie paradigm with the aim of improving compliance and decreasing motion during imaging, a particularly important confound in young children. INSCAPES has been shown to evoke an intermediate level of stimulus-evoked information processing in healthy controls, less so than a movie but more so than rest. However, the effect this may have on resting-state networks in children with migraine has yet to be explored. There is evidence that migraine sufferers are hypersensitive to stimuli [71], therefore visual stimuli may modulate visual networks in children with migraine differently than controls. Indeed, we found differences in visual networks in every comparison, and almost all the identified visual networks. However, as migraine sufferers typically do not habituate to stimuli [72], the gentle colours in INSCAPES may in fact be less stimulating than a black crosshair on a white screen.

## Conclusion

In conclusion, we found alterations in connectivity of multiple networks in children with migraine that were modulated by age and pubertal status. Additionally, we found differences in network connectivity were associated with the position of the child in their migraine cycle. This shows that brain alterations begin early in children with migraine, and these alterations are distinct from those seen in adult migraine. This highlights the need for further investigation into the neural mechanisms of migraine in children specifically, to aid in the development of more effective treatments.

## Data Availability

The datasets generated and analysed during the current study are available from the author on reasonable request.

## Abbreviations

BOLD: Blood Oxygen Level Dependent
DSM-IV: Diagnostic and Statistical Manual of Mental Disorders, fourth edition
CN: Cognitive Network
DMN: Default Mode Network
GIFT: Group ICA of fMRI toolbox
IC: Independent Component
ICA: Independent Component Analysis
MRI: Magnetic Resonance Imaging
HICCUP: Healthy Infants and Children Clinical Research Program
HIT: Headache Impact Test
RCADS: Revised Children’s Anxiety and Depression Scale
PedMIDAS: Pediatric Migraine Disability Scale
SMN: Sensorimotor Network
VN: Visual Network
